# Combination of Two Manipulative Techniques for the Treatment of Cervicogenic Dizziness: A Randomized Controlled Trial

**DOI:** 10.3390/life12071023

**Published:** 2022-07-09

**Authors:** Andoni Carrasco-Uribarren, Pilar Pardos-Aguilella, Silvia Pérez-Guillén, Carlos López-de-Celis, Jacobo Rodríguez-Sanz, Sara Cabanillas-Barea

**Affiliations:** 1 Faculty of Medicine and Health Sciences, Universitat International de Catalunya, 08195 Barcelona, Spain; sperezgu@uic.es (S.P.-G.); carlesldc@uic.es (C.L.-d.-C.); jrodriguezs@uic.es (J.R.-S.); scabanillas@uic.es (S.C.-B.); 2 Faculty of Health Sciences, Universidad de Zaragoza, 50009 Zaragoza, Spain; ppardos@unizar.es

**Keywords:** spine, dizziness, disability, manual therapy, neck, randomized controlled trial

## Abstract

Cervicogenic dizziness is clinically associated with upper cervical spine dysfunctions. It seems that manual therapy decreases the intensity of dizziness in these subjects, but what happens to pain measured by pressure pain threshold (PPT) has not been studied. Purpose: analyze the short-term effects of combination two manipulation techniques protocol in worst dizziness intensity (wVAS), dizziness and cervical disability, upper cervical spine mobility and mechanosensivity of cervical tissue. Methods: Assessor-blinded randomized controlled trial was developed. A total of 40 patients with cervicogenic dizziness were randomly divided into two groups. The experimental group received three treatments consisting of a functional massage and a manipulation technique, and compared with a control group. The wVAS, dizziness handicap inventory (DHI), neck disability index (NDI), UCS mobility, and PPTs were measured. Measurements were made at the baseline, first follow-up 48 h after intervention and second follow-up 1 month after the intervention. Results: at second follow-up wVAS (*p* < 0.001), NDI (*p* < 0.001), DHI (*p* < 0.001), and upper right trapezius (*p* < 0.022) and right suboccipital (*p* < 0.043) PPTs showed a difference between groups in favor of the experimental group. Conclusions: apparently, the proposed intervention protocol decreases the intensity of dizziness and the mechanosensitivity of the cervical tissue and improves the feeling of disability due to neck pain and dizziness.

## 1. Introduction

Dizziness is one of the 10 leading causes of visits to emergency services [1]. In the 2015 retrospective, observational study carried out by otolaryngology specialties in the Spanish population, it was observed that 43 out of every 100,000 inhabitants suffer from dizziness [2]. The vestibular, visual, and somesthetic systems are the main receptors that contribute to our orientation. If one of these receptors registers information that is different from the rest, in that case, dizziness of peripheral origin can occur, which is more common than that of central origin. Of all the types of dizziness of peripheral origin, cervicogenic dizziness is the third most common cause [3].

Cervicogenic dizziness (CGD) is defined as the alteration of the perception of the body’s orientation in the space caused by abnormal activity of the cervical afferents, the deterioration of the somesthetic system causes this affect [4,5]. Clinically, dysfunctions of the upper cervical spine (UCS) are associated with this type of patient [6]. The UCS is essential to the somesthetic system [7], with more than 200 mechanoreceptors per gram of muscle. Many muscle spindles are placed in series, allowing better responses to tensile stimuli [8].

There is a theory that altering the input of these cervical receptors could cause dizziness and imbalance [9,10]. It has been proposed that pain, whether due to a primary or secondary event, could be one of the causes of this afferent input disturbance [11]. Furthermore, it can change muscle coordination and activation patterns [12], altering the cortical representation and modulation of the afferent input [13]. This assumption has been evidenced in healthy subjects after injections to induce pain in the neck [14] as in animals [15,16].

Due to the lack of specific CGD diagnosis test, the diagnosis of this ailment is often made through exclusion [17,18]. Therefore, clinical characteristics are helpful to recognize these subjects. The most important characteristic is a drunkenness/lightheadedness sensation [18], an altered cervical proprioception [19], restricted cervical movement and neck pain [20,21]. Different clinical variables could be helpful when evaluating subjects with neck pain and dizziness. These clinical variables include the Dizziness Handicap Inventory (DHI) [22], posturography [17], local pressure pain threshold (PPT) [11], and the UCS range of motion [23,24].

Manual therapy has been shown as a great treatment for CGD, and this is supported by have a great deal of evidence [25]. Reid et al. conducted several CGD-related studies [22,26,27] comparing cervical mobilization and placebo, and they concluded that manual therapy reduced dizziness and improved range of movement. Generally, dizziness intensity and disability are the primary outcomes measured in regard to CGD. One of the conditions for CGD diagnosis is cervical pain [5,28,29,30]. Thus, many studies also record pain intensity. In these subjects, the pain variable is usually assessed using a visual analogue scale or a numerical pain rating scale. However, no studies have been found looking at the effects of manual therapy on PPT in CGD subjects, although PPTs are a reliable way of assessing pain [31]. Scientific evidence suggests that subjects with CGD may require a local approach to these cervical spine dysfunctions [9]. In our work, C0-1, C1-2, and C2-3 segments were treated. Traction manipulation in the resting position is a technique developed to reduce the risk of adverse events during manipulation, and this technique follows IFOMPT recommendations [32]. This treatment is a high-velocity, low-amplitude thrust technique, performed through a traction movement in resting position of the involved segment [33]. The evidence suggests that traction manipulation protocol improves the disability and the intensity of dizziness perceived by the patient [34,35], but is not known what occurs with the self-perceived neck disability and with neck pain measured by PPTs after this treatment in CGD. A study carried out among patients with cervicogenic headaches using a technique with the same objective but without a high-speed impulse showed that the pressure pain threshold improved after applying the technique [36]. Therefore, a randomized controlled clinical trial is proposed to analyze the short-term effects of a traction manipulation protocol on self-perceived disability, on the intensity of dizziness, cervical mobility, and neck pain measured with PPTs.

## 2. Materials and Methods

A single-blind randomized (simple 1:1) controlled trial with two groups was developed. The study was conducted at the University of Zaragoza in compliance with the Helsinki Declaration of Humana Rights and with local ethics committee (CEICA number: PI15/0230). The registration at Clinicaltrials.gov NCT02772042.

All the patients were referred to our service from the otoneurology service. The otoneurologist excluded other causes of dizziness and performed vestibular function tests. The diagnosis of CGD required the presence of neck stiffness and/or pain, dizziness described as ‘unsteadiness’ triggered by neck movement.

The inclusion criteria for our study were a patient age of over 18 years, a Flexion Rotation Test (FRT) with an asymmetry of 10° between right and left or less than 32° on one side indicating decreased mobility of the CCS, and a passive accessory assessment of the UCS (C0-1, C1-2, C2-3) developed to confirm the presence of dysfunction of this region [37]. The exclusion criteria were as follows: having received cervical treatment in the previous month, the presence of red flags (ligament instability and vascular problems) [32,38], the inability to tolerate FRT, or subjects currently involved in compensations.

The sample size was calculated using the GRANMO 7.12 program based on the worst dizziness intensity and DHI outcomes, obtaining the highest number of subjects (40 subjects, 20 per group using the DHI variable). The sample size was calculated with α risk of 0.05, a two-sided test, and the β risk of 0.20 powered 80%. In a previous pilot study, we saw a common standard deviation of 19 for this questionnaire, and the minimum expected difference for DHI is 18; these data were used to calculate the simple size [34,39]. 

A blinded researcher used Microsoft Excel 2010 to randomize the participants (control group *n* = 20 and treatment group *n* = 20). Another researcher recruited the participants that met the criteria, informed them about the study’s participation and provided them with an informed consent form to sign if they agreed. After that, a researcher made the first assessment. This researcher was blinded to the number sequence and intervention assignment. Another researcher created the assignment of groups without knowing what evaluation was to be performed. To implement the random allocation sequence, sequentially numbered opaque sealed envelopes were used. According to the randomly assigned group, the physiotherapist who applied the treatment was the only individual who was aware of the group to which each participant had been assigned after opening the opaque envelope.

The primary outcome of this study was the worst intensity of dizziness registered by visual analogue scale (wVAS) and DHI, and the secondary outcomes were the cervical passive mobility measured through the FRT, NDI and cervical pain assessed by PPT. The measurements were registered at baseline (T0), at 48 h post-intervention (T1), and at one month following the last intervention (T2).

Self-perceived disability was recorded using the wVAS, DHI and NDI questionnaire and intensity of dizziness.

The dizziness intensity was registered with a VAS, a valid and reliable tool for measuring dizziness intensity (reliability test-retest of this VAS is r = 0.85–0.96) [40]. A continuous vertical line of 100 mm was anchored by two verbal descriptors (“no dizziness” and “worst imaginable dizziness”), and each end of the line had a mark; the subjects had to mark on the line the worst perceived dizziness since the last appointment (wVAS). Although it is a subjective and individual scale of the patient, it allows us to register the intensity of dizziness quickly, and its use in the clinic is widespread. According to the Fong et al. [41] review, its use in the scientific community is widespread.

The DHI Is a highly reliable and responsive tool [42,43]. This questionnaire is validated to Spanish and shows a high test–retest reliability (ICC = 0.98). This version is comprised of 25 questions designed to assess patients’ functional, emotional, and physical limitations on a three-point scale [42]. The highest available score is 100, indicating the maximum level of self-perceived handicap (0–30 “low handicap”, 30–60 “moderate handicap” and +60 “severe handicap”) [44].

The Spanish version of the NDI was used to measures self-perceived neck disability. With a high test-retest reliability (ICC = 0.978) [45], this questionnaire consists of 10 items (7 of them of daily living activities, 2 related of pain and 1 of concentration). Each question is scored from 0 to 5. The maximum score of the questionnaire is 50 points, with the highest scores being those associated with a greater degree of self-perceived disability. The use of this questionnaire has been proposed to monitor adverse events after treatment [38]. 

The test used to measure the range of motion of the UCS was FRT, which has been reported to be a valid and reliable measurement of UCS [46,47], and that it has shown excellent test-retest reliability (ICC = 0.93) [47]. For the measurement of FRT, the participant was positioned in supine. The cervical spine was flexed to the maximum degree and rotated from the left side to the right side three times; this method of measurement was described in previous studies [46,47]. After observing that the FRT was positive, it was registered as a more restricted side (FRT MRS) and less restricted side (FRT LRS) [48].

To measured cervical PPT, a digital algometer was used (Sonomedic SenseLab, AB, Farsta, Sweden), with an almost perfect inter-rater (ICC = 0.815–0.940) and within-session test-retest (ICC = 0.854–0.906) reliability [18], in patients with cervical pain and dizziness. The pressure was applied at the rate of 1 kg/cm3/s perpendicularly to the skin with a round surface area of 1 cm^2^. The participants were in a supine position, and the PPT was assessed bilaterally over 3 points: C2-3 zygapophyseal joint, suboccipital muscles, and upper trapezius. Patients were instructed to press the button of the digital algometer at the precise moment that the pressure sensation turned into pain.

Furthermore, at T0, fear of movement was recorded with the Tampa Kinesiophobia Scale (TSK-11).

The measurements were carried out at the University of Zaragoza. The researcher who recorded the outcome measures at baseline, at T1 and T2 follow-up was a specialist in manual therapy, with more than 10 years of experience in physical therapy. This researcher was blinded to the allocation group of each participant throughout the process. Another physiotherapist specializing in manual therapy and with more than 10 years of experience performed the intervention protocol.

The experimental and control group received three interventions on alternate days. The treatment took 11 min and was divided into three sections. 

In the first section (pre-manipulative section), the participant was in a supine position. The techniques were applied to improve the subject’s state of relaxation and to assess the security of the technique. The pre-manipulative techniques performed were based on suboccipital massage according to the treatment protocol.

**Figure 1:**
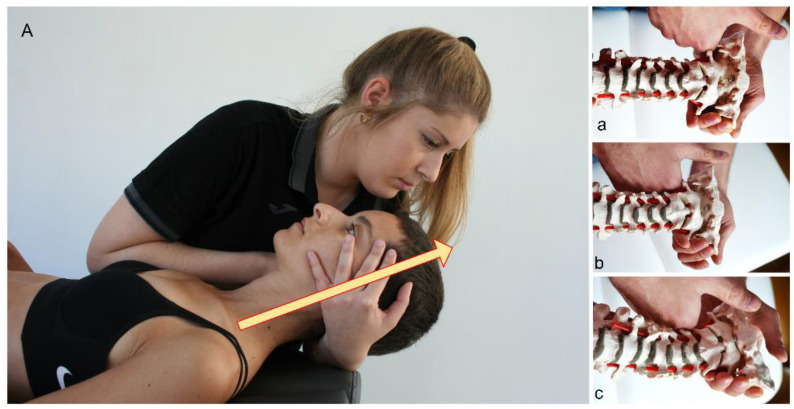
Second section of the treatment protocol (manipulation section). (**A**): Real technique. (**a**–**c**): Model technique. (**Aa**), HVLA C0-1. (**Ab**), HLVA C1-2. (**Ac**), HVLA C2-3. HVLA, high-velocity low-amplitude traction manipulation.

In the second section (manipulation section), the C0-1, C1-2, and C2-3 joints were assessed after the application of the pre-manipulative technique and before the application of the manipulation. The manipulation technique was applied only to the joints showing limited accessory movement and altered end feeling. The manipulation was a technique that utilized high velocity and low amplitude, and this was performed in the segments C0-1 (Figure 1Aa), C1-2 (Figure 1Ab) or C2-3 (Figure 1Ac) in the direction of traction, with the head in a neutral position (Figure 1). 

In the last section (post-manipulation section), the patient remained supine for 1 min, and it was suggested that the patient slowly entered a sitting position.

The control group remained supine for the same period as the experimental group. After the study, the control group subjects were offered the treatment performed on the experimental group. This fact was only communicated following the completion of the study and analysis of what occurred within the experimental group.

This protocol has been previously described by the literature as a strategy to improve and promote patient safety [35]. However, to be sure of the safety of the intervention protocol, the participants were asked to contact the research group as soon as possible if they perceived a worsening of their condition or other symptoms related to the research.

The statistical analysis was carried out with the SPSS 20.0 package (IBM, Armonk, NY, USA). There was no loss follow up in the study. The mean and standard deviation were calculated for each variable. The Shapiro–Wilk test was used to determine a normal distribution of quantitative data and a chi-square test was done for the DHI and gender qualitative variables (*p* > 0.05). To analyze between-group difference, the student t-test was used for quantitative data and for the qualitative data, Fisher test. Within-group differences were analyzed using repeated measures of ANOVA and one-way ANOVA. If the assumption of sphericity was violated, Greenhouse–Geisser correction was utilized for interpretation. The significance level was set at *p* < 0.05. In addition, the effect sizes were calculated using Cohen’s d coefficient. Effect sizes for quantitative variables were calculated using Cohen’s d coefficient. Effect sizes for qualitative variables (DHI) was calculated with the V’s Cramer. An effect size >0.8 was considered large; about 0.5, intermediate; and <0.2, small [49]. 

## 3. Results

**Figure 2:**
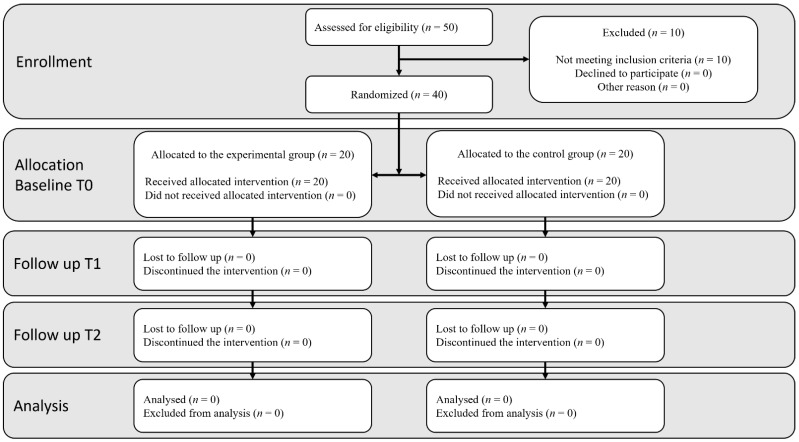
CONSORT. (Consolidated Standards of Reporting Trial) flow diagram.

A total of 50 participants were recruited; 80% of the subjects met the selection criteria and agreed to participate (32 female and 8 males; 54 years ± 14.09 years). A total of 20 subjects were randomly assigned to each group and received the intended treatment. The DHI showed low handicap in 35% of the subjects at the experimental group and in 30% of the control group, moderate handicap in 50% of the subjects at the experimental group and 60% at the control group and severe handicap in 15% of the subjects at the experimental group and 10% in the control group. Figure 2 shows the flow chart, and the demographic data at baseline were presented at Table 1.

### 3.1. wVAS

In the experimental group, the worst intensity of dizziness was significantly lower after the intervention (*p* < 0.001, d = 2.43), and at T2 follow-up (*p* < 0.001, d = 1.64). At T2 follow up were significant difference between group in favor the experimental group (*p* < 0.001) with large effect size (d = 1.28) (Table 2).

### 3.2. DHI

**Figure 3:**
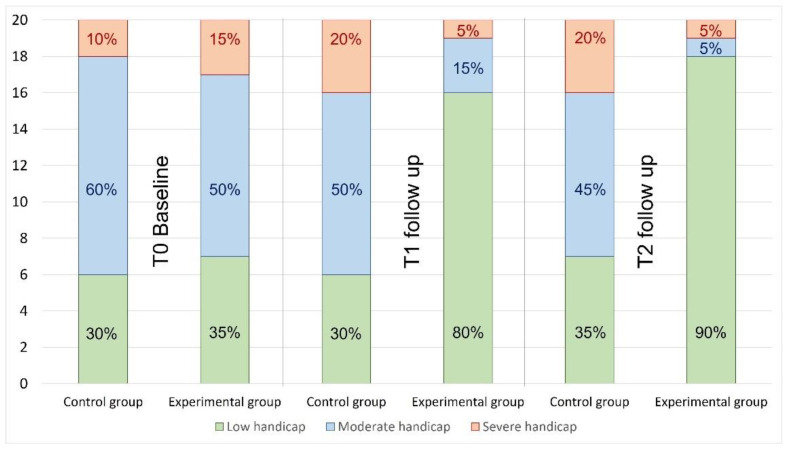
Dizziness Handicap Inventory during the study in control and experimental group.

In the experimental group, the subjects who perceived severe dizziness handicap decreased from 3 to 1 at T1 and T2 follow ups. In the same line, the subjects that felt moderate dizziness handicap decreased from 12 to 3 between T0 to T1 follow ups, and only 1 felt moderate disability. In the control group, the subjects who perceived severe dizziness handicap increased in 50% from 2 to 4 between T0 and T1 follow up and remained equal in T2 follow up, and the case of moderate dizziness handicap decreased in 5% between T0 and T2 follow up, from 10 to 9 (Figure 3). 

The analysis between groups showed difference in favor of the experimental group at T1 follow up (*p* < 0.005, V = 0.503) and at T2 follow-up (*p* < 0.001, V = 0.571). 

### 3.3. NDI

In the experimental group, the disability perceived by the patient was significantly lower after the intervention at T1 follow-up (*p* < 0.001, d = 0.53). At T2 follow-up, the reduction was of 4.5 point (*p* < 0.002, d = 0.73). In the control group also decreased, but only in 1.2 point at T1 follow-up and in 1 point at T2 follow-up (Table 2). Between groups, significant differences were found at T2 follow-up (*p* < 0.012, d = 0.94) (Table 2).

### 3.4. UCS Movement (FRT)

At T1 and T2 follow-ups, an increase in the passive cervical range of movement was observed in the experimental group. This group showed a significant improvement at T1 follow-up (*p* < 0.001, d = 2.21) and at T2 follow-up (*p* < 0.001, d = 2.23) (Table 2). For the FRT LRS, the experimental group increased the range of movement in both moments at the T1 follow-up (*p* < 0.001, d = 1.35) and to T2 follow-up (*p* < 0.002, d = 1.09). The passive range of motion of the upper cervical spine hardly varied during the period evaluated in the control group. In the comparison between groups for the FRT for MRS, the results are favorable to the experimental group in T1 follow-up (*p* < 0.001, d = 1.36) and in T2 follow-up (*p* < 0.001, d = 1.23).

### 3.5. Pressure Pain Threshold

After the intervention protocol, statistically significant results between groups were observed in the upper right trapezius at T1 follow-up (*p* < 0.006, d = 0.35) and at T2 follow-up (*p* < 0.022, d = 0.22) and in the right suboccipital (*p* < 0.043, d = 0.56) (Table 3). The tissue mechanosensitivity was reduced in almost all points for both groups, achieving only statistically significant results in the experimental group in the right upper trapezius (*p* < 0.022, d = 0.46) and left upper trapezius (*p* < 0.035, d = 0.26) for T1 follow-up. For the T2 follow-up, there were observed statistically significant results in the right upper trapezius (*p* < 0.016, d = 0.43), left upper trapezius (*p* < 0.009, d = 0.37) and right suboccipital (*p* < 0.018, d = 0.46). 

No adverse effects were recorded during the intervention protocol either in any of the follow-ups.

## 4. Discussion

Through this study, it can be observed that the proposed intervention protocol in patients with CGD improved the disability, the intensity of dizziness, and the passive ROM of the UCS. After the intervention protocol as well as in the 1-month follow-up, it also decreased the mechanosensitivity of the tissue measured with PPT. 

The reduction of dizziness intensity in the experimental group is larger than the MCID in the T1 and T2 follow-ups. The reduction was 48.80 mm at T1 and 37.05 mm at T2 follow-up, and the MCID was quantified in 20 mm [27,30]. Normally the analogical visual scale records the average dizziness perceived by the patient. However, in our case, we found it interesting to know the worst intensity perceived by the patient. Only two studies have been found that collect the wVAS. One of these is Heikkilä et al. [50], who conducted a pilot study to compare acupuncture, manipulation therapy and an NSAID-gel (ketoprofen), performing four treatment session without differences between groups, and the acupuncture showed a decrease in the wVAS. The other is a case-control study [34] that observed the immediate effects (just after the intervention) of three treatment sessions of the same protocol as in our study; they observed a statistically significant improvement but with a reduction in the intensity of dizziness that was lower than ours.

The DHI questionnaire Is the most used tool to record self-perceived disability [41,51]. The patients who recorded a greater disability level in the questionnaire showed greater functional impairment [52]. Manual therapy is effective in improving the quality of life in this type of pathology [25]. The improvement in quality of life is reflected in the number of patients that perceived less disability dizziness in the DHI questionnaire, as seen with the experimental group of the present study. In the experimental group, the subjects who perceived a severe handicap went from 3 to 1 in the T1 follow-up, and in the T2 follow-up, the case of those who perceived a moderate handicap was more marked, since 9 people reduced it in the T2 follow-up. Reid et al. [28] compared two manual therapy techniques with a placebo group. One of the groups was treated using mobilization techniques described by Mulligan (SNAGs), another with techniques described by Maitland (PJM), and the last was a placebo group (Laser). Each group received an average of four treatment sessions divided over 6 weeks. As such, so they received one more treatment session than our experimental group. The group with mobilizations of SNAGs improved 8.6 points, while the group with mobilizations of PJM improved by 15.2 points after the intervention. These results are very similar to those found in the present study [28,37]. The fact that the intensity of dizziness has decreased in the experimental group subjects could be the reason for the decrease in the DHI score and, therefore, for less self-perceived disability.

The NDI questionnaire is a poorly studied outcome measure in subjects with CGD. A change in score of 10% or more is stated to be clinically relevant, and a decrease of 5 points indicates the least detectable change [53]. In our study we obtained, in both follow-ups in the experimental group, a decrease of more than 10% with respect to the initial value in the questionnaire. Strunk et al. [54] conducted a single-group study, with two sessions per week for 8 weeks. The approximate duration of the treatment was 15 to 20 min. The intervention included various physiotherapy techniques (manipulation, mobilization, muscle treatment, heat and cold). They obtained a reduction of 3.41 points after 8 weeks of treatment. Our results are very similar, with a decrease of 3.05 points at T1 and 4.45 points in T2 follow-ups. Mínguez-Zuazo et al. [55] performed an intervention that focused on patient education and therapeutic exercises. They observed a mean improvement of 5.14 points, this result being somewhat higher than that obtained in our study. This study is the only one that we have found that proposes the combination of therapeutic exercise and patient education; the combination of these seems effective for treating different musculoskeletal dysfunctions [56].

To report adverse cases and processes after the application of treatments based on manual therapy, use of the DHI, NDI and the VAS is proposed. It should be noted that, in our study, none of the participants presented adverse effects in any of the follow-up periods [38].

Improvements in pressure mechanosensitivity were found in all points analyzed in the experimental group, and the control group also showed some improvements. There was a difference favoring the experimental group in the right and left upper trapezius in the T1 and T2 follow-ups. Additionally, the right suboccipital muscle showed these results at the T2 follow-up. We have not found RCTs that analyze PPTs in subjects with CGD. However, Knapstad et al. [31] found that subjects with cervical pain and dizziness have a lower pressure tolerance. The percentage of change that is considered the MCID for pressure pain thresholds is 15% [57]. The experimental group showed higher changes than 15% at T1 follow-up for right upper trapezius (30%), right C2-3 joint (18%), and right (23%) and left (22%) suboccipital muscle and at T2 follow-up for right (29%) and left (27%) upper trapezius, left C2-3 joint (32%), and right (27%) and left (22%) suboccipital muscle. The control group only showed these changes for the left C2-3 joint at T1 follow-up and left suboccipital muscle (20%) at T2 follow-up. The study by Knapstad et al. [31] observed an inverse association between this variable and body stability, so we can think that the decrease in cervical tissue mechanosensitivity could have improved body stability, which in turn could have improved the perceived sensation of dizziness.

The upper cervical dysfunction could be one of the causes of the CGD [6,58]. Few studies have measured the range of motion of the upper cervical spine [23,35]. The FRT is a validated test to measure the range of movement of the UCS [47,59,60], and this can be useful in the diagnosis of CGD [24]. The range of motion of the upper cervical spine improved in the experimental group in both follow-ups. On the contrary, the control group remained practically the same. Jung et al. [23] performed the FRT assessment in patients with cervicogenic dizziness. This author suggested personalized treatment based on the findings that each patient presented. In their study, although the FRT was quantified as positive or negative, it is intuited that the subjects who improved in the FRT also showed improvement in the intensity of dizziness.

There is a discussion about the origin of cervicogenic dizziness. The great existence of mechanoreceptors in the upper cervical spine and the alteration of the information received from this region is one of the most accepted theories. Pain in this region could modulate proprioceptive input [61]. The activated mechanoreceptors in the lesion area would produce a nociceptive response, causing a temporary peripheral sensitization. This increases the receptor thresholds, giving rise to painful responses to non-harmful stimuli [62,63]. The generation of pain from mechanoreceptors, which generally generate harmless responses, could increase the nociceptive input, and decrease the non-nociceptive input, altering the somatosensory input [63]. This seems to occur in acute and chronic patients, although somatosensory alteration is greater in those with longer pathologies [62,64,65].

Current evidence suggests that manual therapy produces immediate analgesia through neurophysiological mechanisms. In this case, the proposed intervention produces a mechanical stimulus in the cervical spine. This technique would produce an analgesic effect by activating pain inhibitors [66,67,68]. Therefore, it has been observed that manual therapy has neurophysiological effects that allow us to relieve pain and, in this specific case, dizziness. There are studies with similar techniques that have similar results to ours [69,70,71,72]. However, in most cases, they did not mention whether or not these techniques follow the IFOMPT safety recommendations for the treatment of the UCS. This institution made a series of recommendations to reduce the adverse effects that can occur after treatment of the upper cervical spine [32,38]. The intervention protocol follows the IFOMPT safety recommendations, so we believe it is a safe treatment with great potential for clinical use.

After the intervention protocol, an improvement in the range of motion of the UCS and a decrease in pain was observed. These findings have also been observed in other studies with the application of similar treatments. The decrease in pain produced by the intervention protocol could decrease the nociceptive input, normalizing the mechanoreceptive input and producing a decrease in dizziness while normalizing the range of motion. More studies are needed to determine the hypoalgesic effects of manual therapy interventions in patients with CGD.

Although the inclusion of a control group was a potential strength of the current controlled clinical trial, we must recognize the presence of various limitations.

Our study shows the short-term effects of the proposed intervention protocol. Therefore, short- and medium-term effects cannot be guaranteed or deduced. Placebo effects have been investigated in other studies in patients with CDG, and the placebo effect has never been greater than the effect of the techniques performed. However, in our study, we did not perform any treatment on the control group. Thus, we cannot assure that the effect of our techniques is not due to this cause. Therefore, it would be necessary to carry out a similar study to compare this with a placebo group. In the reviewed literature, no study with a control group was found. The most similarity to the control group was found in a study that kept one of its groups on the waiting list for 8 weeks [29]. The present study was carried out with a control group since it is unknown if the symptoms fluctuate, especially when evaluations could modify them. However, no change was found for consideration during the study period. Although our study only evaluated the passive range of motion with the FRT, it has been observed that some psychological factors, such as catastrophizing and kinesophobia, can alter the active range of motion of the cervical spine [73]. As such, future studies should consider these variables.

Therefore, more studies are needed to make a correct diagnosis of these subjects with CGD. In addition, it would be interesting to know the results of a local approach to the cervical spine and study the immediate, short-term, and medium-term effects.

## 5. Conclusions

The proposed intervention protocol appears to improve dizziness intensity, perceived disability from neck pain, and dizziness at follow-up T1 and T2. Additionally, it could help increase the range of motion of the upper cervical spine at both follow-ups. Finally, after the intervention, we have found an improvement in the mechanosensitivity of the right upper trapezius and the right suboccipital muscle that had not been described until now.

## Figures and Tables

**Table 1:** Baseline demographic and clinical characteristics for each group.

	Experimental Group (*n* = 20)	Control Group (*n* = 20)	*p* Value
Gender (M; F)	4M;16F	4M;16F	0.999
Age (years)	55.90 (11.96)	52.10 (16.03)	0.401
Weight (kg)	65.90 (11.96)	66.90 (11.56)	0.730
Height (cm)	163.30 (8.53)	162.40 (7.79)	0.790
wVAS	74.45 (17.34)	70.94 (23.48)	0.585
NDI	12.45 (5.96)	14.60 (5.44)	0.231
TSK-11	24.20 (5.72)	24.45 (6.82)	0.901
DHI			
Severe	3 (15%)	2 (10%)	0.795
Moderate	10 (50%)	12 (60%)	
Low	7 (35%)	6 (30%)	

Abbreviations: M, Male; F, Female; kg, Kilograms; cm, centimeters; wVAS, worst visual analogue scale; NDI, Neck Disabilty Index; TSK-11: Tampa Kinesophobia Scale; DHI, Dizziness Handicap Inventory.

**Table 2:** Differences within and between group during the study in wVAS, NDI and FRT range of motion.

Group	Baseline	Follow-Up	Within Group	Between Group	Follow-Up	Within Group	Between Group
	T0	T1	T0-T1	T0-T1	T2	T0-T2	T0-T2
wVAS							
**Control** **group**	70.85 ± 23.48	61.20 ± 25.89	*p* < 0.334	*p* < 0.001 d = 1.46	67.75 ± 20.38	*p* < 0.999	*p* < 0.001 d = 1.28
			d = 0.39			d = 0.14	
**Experimental** **group**	74.45 ± 17.34	25.65 ± 22.50	*p* < 0.001		37.05 ± 27.08	*p* < 0.001	
			d = 2.39			d = 1.64	
NDI							
**Control** **group**	14.60 ± 5.44	13.40 ± 6.84	*p* < 0.285	*p* < 0.064 d = 0.64	13.60 ± 5.56	*p* < 0.625	*p* < 0.012 d = 0.94
			d = 0.19			d = 0.18	
**Experimental** **group**	12.25 ± 5.96	9.35 ± 5.77	*p* < 0.001		7.95 ± 6.39	*p* < 0.002	
			d = 0.53			d = 0.73	
FRT Most restricted side							
**Control** **group**	18.65 ± 6.96	19.63 ± 8.04	*p* < 0.999	*p* < 0.001 d = 1.36	20.83 ± 8.26	*p* < 0.686	*p* < 0.001 d = 1.23
			d = 0.13			d = 0.29	
**Experimental** **group**	17.27 ± 4.61	29.40 ± 6.23	*p* < 0.001		30.03 ± 6.66	*p* < 0.001	
			d = 2.21			d = 2.23	
FRT Less restricted side							
**Control** **group**	23.40 ± 7.72	20.98 ± 7.70	*p* < 0.558	*p* < 0.001 d = 1.43	21.66 ± 8.48	*p* < 0.944	*p* < 0.001 d = 1.03
			d = 0.31			d =0.22	
**Experimental** **group**	22.13 ± 6.75	31.01 ± 6.41	*p* < 0.001		29.78 ± 7.27	*p* < 0.002	
			d = 1.35			d = 1.09	

Abbreviations: wVAS, worst visual analogue scale; NDI, Neck Disability Index; FRT, Flexion Rotation Test.

**Table 3:** Differences within and between group during the study in pressure pain threshold.

Group	Baseline	Follow-Up	Within Group	Between Group	Follow-Up	Within Group	Between Group
	T0	T1	T0-T1	T0-T1	T2	T0-T2	T0-T2
Upper Trapezius (R) (kPa)							
**Control** **group**	188.90 ± 66.86	178.45 ± 100.14	*p* < 0.999	*p* < 0.006 d = 0.35	191.75 ± 84.20	*p* < 0.999	*p* < 0.022 d = 0.22
			d = 0.12			d = 0.04	
**Experimental** **group**	166.55 ± 98.53	217.10 ± 122.25	*p* < 0.022		214.90 ± 126.60	*p* < 0.016	
			d = 0.46			d = 0.43	
Upper Trapezius (L) (kPa)							
**Control** **group**	174.75 ± 78.46	173.10 ± 93.47	*p* < 0.999	*p* < 0.086 d = 0.34	187.20 ± 101.84	*p* < 0.999	*p* < 0.134 d = 0.32
			d = 0.02			d = 0.14	
**Experimental** **group**	177.65 ± 125.16	208.55 ± 112.65	*p* < 0.035		225.85 ± 137.14	*p* < 0.009	
			d = 0.26			d = 0.37	
C2-3 (R) (kPa)							
**Control** **group**	139.15 ± 52.42	146.20 ± 60.93	*p* < 0.568	*p* < 0.273 d = 0.43	150.50 ± 53.59	*p* < 0.579	*p* < 0.639 d = 0.38
			d = 0.12			d = 0.21	
**Experimental** **group**	152.30 ± 97.02	179.85 ± 91.67	*p* < 0.408		172.60 ± 62.56	*p* < 0.738	
			d = 0.26			d = 0.25	
C2-3 (L) (kPa)							
**Control** **group**	129.25 ± 54.98	150.25 ± 70.93	*p* < 0.410	*p* < 0.838 d = 0.25	139.85 ± 59.71	*p* < 0.999	*p* < 0.110 d = 0.74
			d = 0.33			d = 0.19	
**Experimental** **group**	152.35 ± 86.11	169.75 ± 84.19	*p* < 0.399		201.55 ± 102.43	*p* < 0.071	
			d = 0.20			d = 0.52	
Suboccipital (R) (kPa)							
**Control** **group**	156.70 ± 59.68	160.25 ± 66.18	*p* < 0.999	*p* < 0.070 d = 0.35	157.90 ± 59.67	*p* < 0.999	*p* < 0.043 d = 0.56
			d = 0.07			d = 0.02	
**Experimental** **group**	157.70 ± 97.81	193.90 ± 120.61	*p* < 0.075		201.05 ± 91.94	*p* < 0.018	
			d = 0.33			d = 0.46	
Suboccipital (L) (kPa)							
**Control** **group**	132.80 ± 48.77	142.55 ± 188.05	*p* < 0.064	*p* < 0.143 d = 0.30	159.45 ± 59.12	*p* < 0.999	*p* < 0.773 d = 0.42
			d = 0.71			d = 0.14	
**Experimental** **group**	153.20 ± 125.53	188.05 ± 102.29	*p* < 0.999		187.45 ± 73.25	*p* < 0.001	
			d = 0.30			d = 1.64	

Abbreviations: R, Right; L, Left; kPa, kilopascal.

## Data Availability

The datasets analyzed during the current study are available from the corresponding author on reasonable request. All data analyzed during this study are included in this published article.
